# The Isl1-Lhx3 Complex Promotes Motor Neuron Specification by Activating Transcriptional Pathways that Enhance Its Own Expression and Formation

**DOI:** 10.1523/ENEURO.0349-16.2017

**Published:** 2017-03-30

**Authors:** Madalynn Erb, Bora Lee, So Yeon Seo, Jae W. Lee, Seunghee Lee, Soo-Kyung Lee

**Affiliations:** 1 Papé Family Pediatric Research Institute, Department of Pediatrics, Oregon Health and Science University, Portland, OR 97239; 2 Vollum Institute, Oregon Health & Science University, Portland, OR 97239; 3 College of Pharmacy and Research Institute of Pharmaceutical Sciences, Seoul National University, Seoul, Korea

**Keywords:** Isl1, Isl1-Lhx3, Lhx3, LMO4

## Abstract

Motor neuron (MN) progenitor cells rapidly induce high expression of the transcription factors Islet-1 (Isl1), LIM-homeobox 3 (Lhx3), and the transcriptional regulator LMO4, as they differentiate. While these factors are critical for MN specification, the mechanisms regulating their precise temporal and spatial expression patterns are not well characterized. Isl1 and Lhx3 form the Isl1-Lhx3 complex, which induces the transcription of genes critical for MN specification and maturation. Here, we report that *Isl1*, *Lhx3*, and *Lmo4* are direct target genes of the Isl1-Lhx3 complex. Our results show that specific genomic loci associated with these genes recruit the Isl1-Lhx3 complex to activate the transcription of *Isl1*, *Lhx3*, and *Lmo4* in embryonic MNs of chick and mouse. These findings support a model in which the Isl1-Lhx3 complex amplifies its own expression through a potent autoregulatory feedback loop and simultaneously enhances the transcription of *Lmo4*. LMO4 blocks the formation of the V2 interneuron-specifying Lhx3 complex. In developing MNs, this action inhibits the expression of V2 interneuron genes and increases the pool of unbound Lhx3 available to incorporate into the Isl1-Lhx3 complex. Identifying the pathways that regulate the expression of these key factors provides important insights into the genetic strategies utilized to promote MN differentiation and maturation.

## Significance Statement

The precise temporal and spatial regulation of transcription factor expression is critical for embryos to generate the appropriate number and variety of motor neurons (MNs). This process dictates the formation of motor circuits, which regulate coordinated movement and homeostasis. When MN specification is impaired, it leads to serious medical conditions such as spinal muscular atrophy. Understanding MN development is crucial for effectively treating pediatric MN disorders and neurodegenerative disorders, such as amyotrophic lateral sclerosis. Here, we show that three essential factors for MN development, Islet-1 (Isl1), LIM-homeobox 3 (Lhx3), and LMO4, are induced directly by the Isl1-Lhx3 complex. Characterizing the pathways that direct the expression of these factors provides key insights into the genetic mechanisms that regulate MN development.

## Introduction

Combinatorial expression of specific transcription factors establishes discrete progenitor domains, in the embryonic spinal cord, which each generate distinct types of neurons (Jessell, 2000; Lee and Pfaff, 2001). The p0-p3 domains generate ventral interneurons and the pMN domain generates motor neurons (MNs; Jessell, 2000; Lee and Pfaff, 2001). While the signaling cascades that establish the pMN domain are well characterized, the mechanisms that promote the initiation and maintenance of transcription factor expression in developing MNs remain unclear. As these factors are critical for MN specification and diversification, understanding the pathways that regulate their expression will provide important insights into this process.

Immediately prior to differentiation, pMN cells express two LIM-homeodomain transcription factors, Islet-1 (Isl1) and LIM-homeobox 3 (Lhx3; Mizuguchi et al., 2001; Roy et al., 2012). Both proteins contain two LIM domains that facilitate protein-protein interactions, as well as a single homeodomain, which binds DNA (Bhati et al., 2008, 2012). When coexpressed, Isl1 and Lhx3 interact with each other and with nuclear LIM interactor (NLI) to form a hexameric transcription complex, called the Isl1-Lhx3 complex, with a 2:2:2 stoichiometry (Thaler et al., 2002). When Lhx3 is expressed in the absence of Isl1, as is the case in developing V2 interneurons, Lhx3 and NLI form the tetrameric Lhx3 complex, with a 2:2 stoichiometry (Thaler et al., 2002). The Isl1-Lhx3 complex primarily functions through binding to the long hexamer response element (HxRE-Long), and the short hexamer response element (HxRE-Short) (Lee et al., 2008, 2013). The HxRE-Short is also known as the tetramer response element (TeRE), and it is also recognized and bound by the tetrameric Lhx3 complex (Lee et al., 2008). Binding of the Isl1-Lhx3 complex to HxRE-Long and HxRE-Short elements activates the transcription of genes that are essential for MN specification such as *Hb9*, and genes that are required for cholinergic neurotransmission, such as *VaCHT* (Thaler et al., 2002; Lee and Pfaff, 2003; Lee et al., 2012).

To efficiently transition from a progenitor state to a terminally differentiated state, pMN cells must rapidly upregulate and maintain the expression of Isl1 and Lhx3. Deletion of Isl1 or Lhx3, or disruption of Isl1-Lhx3 complex assembly, severely impairs MN specification (Pfaff et al., 1996; Sharma et al., 1998; Thaler et al., 2002; Song et al., 2009; Liang et al., 2011). Following MN specification and migration, Isl1 expression is maintained in many MN subtypes, but Lhx3 expression is only maintained in medial motor column (MMCm) neurons (Tsuchida et al., 1994; Rousso et al., 2008). Despite recent progress characterizing the spatial and temporal patterns of gene expression in differentiating MNs, the genetic mechanisms that direct differentiating MNs to induce high levels of Isl1 and Lhx3 transcription during MN specification, and the mechanisms utilized to maintain high levels of Isl1 and Lhx3 expression in MMCm neurons remain unclear.

Here, we report that the Isl1-Lhx3 complex binds two distinct genomic regions downstream of *Lhx3*, as well as a known Isl1 enhancer (Uemura et al., 2005; Kim et al., 2015). Interestingly, we also found a binding site located in the second intron of *Lmo4*, which encodes LIM only protein 4 (LMO4). LMO4 is expressed in embryonic MNs, and is important for inhibiting the formation of the Lhx3 complex, indirectly increasing the probability that Lhx3 will incorporate into the Isl1-Lhx3 complex (Lee et al., 2008; Song et al., 2009). Using GFP-reporter studies and embryonic chick neural tube electroporation, we found that each of these Isl1-Lhx3 binding sites act as MN-specific enhancers and each is activated by the Isl1-Lhx3 complex. Therefore, our results show that the Isl1-Lhx3 complex activates two distinct transcription pathways in parallel to enhance its own expression and formation during MN development. First, a positive autoregulatory loop amplifies the expression of the complex’s key components, Isl1 and Lhx3. Second, the Isl1-Lhx3 complex activates the expression of LMO4, which indirectly promotes Lhx3 incorporation into the Isl1-Lhx3 complex.

## Materials and Methods

All embryo experiments in this study were performed without determining the sex of each embryo.

### Chromatin immunoprecipitation (ChIP)-quantitative PCR (qPCR) assays

We isolated spinal cords from E12.5 mouse (ms) embryos. Spinal cords from 5-12 embryos were combined for each ChIP reaction with a specific antibody. Antibodies used for immunoprecipitation were rabbit (rb) anti-Isl1/2 (kindly provided by Tom Jessell, Columbia University; Tsuchida et al., 1994), rb anti-Lhx3 (Abcam ab14555), and nonspecific rb IgG. The tissues were dissociated completely before the ChIP process. Next, cells were washed with buffer I (0.25% Triton X-100, 10 mM EDTA, 0.5 mM EGTA, and 10 mM HEPES, pH 6.5) and buffer II (200 mM NaCl, 1 mM EDTA, 0.5 mM EGTA, and 10 mM HEPES, pH 6.5) sequentially. Then, cells were lysed with lysis buffer (0.5% SDS, 5 mM EDTA, 50 mM Tris·HCl, pH 8.0, and protease inhibitor mixture) and were subjected to sonication for DNA shearing. Next, cell lysates were diluted 1:10 in ChIP buffer (0.5% Triton X-100, 2 mM EDTA, 100 mM NaCl, 50 mM Tris·HCl, pH 8.0, and protease inhibitor mixture) and, for immunoclearing, were incubated with IgG and protein A agarose beads for 1 h at 4°C. Supernatant was collected after quick spin and incubated with IgG or the afore-mentioned antibodies and protein A agarose beads overnight at 4°C. After pull-down of chromatin/antibody complex with protein A agarose beads, the beads were washed with TSE I (0.1% SDS, 1% Triton X-100, 2 mM EDTA, 20 mM Tris·HCl, pH 8.0, and 150 mM NaCl), TSE II (same components as in TSE I except 500 mM NaCl), and buffer III (0.25 M LiCl, 1% Nonidet P-40, 1% deoxycholate, 1 mM EDTA, and 10 mM Tris·HCl, pH 8.0) sequentially for 10 min at each step. Then the beads were washed with TE buffer three times. Protein/chromatin complexes were eluted in elution buffer (1% SDS, 1 mM EDTA, 0.1 M NaHCO_3_, and 50 mM Tris·HCl, pH 8.0) and de-crosslinked by incubating at 65°C overnight. Eluate was incubated at 50°C for more than 2 h with proteinase K. Next, DNA was purified with phenol/chloroform and DNA pellet was precipitated by ethanol and resolved in water. The purified final DNA samples were used for qPCRs using the SYBR green kit (11762-500, Invitrogen) and CFX Connect (Bio-Rad). The total input was used for normalization. All ChIP experiments were repeated independently at least three times. Data are represented as the mean of duplicate or triplicate values obtained from representative experiments; error bars represent SEM. Following immunoprecipitation, qPCR was performed to detect peaks using the following primers. Lhx3-Peak-A Fwd: GGTCTGCCTCCCGTAAAACT and Rev: CACCATCAATGCTTTGTTCAG, Lhx3-Peak-B Fwd: CAATGCAGGGTGACCTGG and Rev: GTGGGATTGACTGGGGTC, Isl1-Peak Fwd: CTGCCACTCCACTTAATAACCTAA and Rev: ATGGACACACCAGCTGGATAAATC, and LMO4-Peak Fwd: ATCACTCGAGGACGTGGGTCCCTTTAAGATCC and Rev: CTGAGTCGACGGATTCTGCCTCCTCTCCTC.

### *In ovo* electroporation

Electroporation was performed in HH st12-14 chick embryos, by injecting DNA into the embryonic neural tube. A square pulse electroporater was used to apply five pulses, 25 V, 50 ms with 1 s between each pulse across the neural tube. Enhancers were cloned into PBS-miniCMV-eGFP or SP72-TATA-eGFP reporter plasmids. Lhx3-Peaks and the LMO4-Peak were cloned from the mouse genome, and the Isl1-Peak was cloned from the human genome. Embryos were injected with 2.5 µg/µL of reporter construct and 1.75 µg/µL of LacZ or 1.75 µg/µL of Isl1-Lhx3 expression construct. Embryos were harvested and processed for immunolabeling 3 d postelectroporation (3DPE), at HH st25. Images are representative of electroporations from multiple embryos.

### Cloning of Isl1-Lhx3 ChIP-Seq loci

The Isl1-Lhx3 ChIP-Seq peaks were constructed using the following primer sets. We have also listed the sequences for wt HxRE sequences and the sequences for mutated HxREs. Mutations were introduced using PCR. *Lhx3-Peak-A (ms)* (chr2:26194774-26194788): Fwd-CTAGAGGTAGCCAAGGCC and Rev-TGGAGAGGGCTAGCCAC. Hx-L-wt: CATTTTAACTAATGG ΔHx-L: CGCGGCCGCAGCCGG. Hx-S-wt: CTAATTAAA ΔHx-S: CGGCCGCAA. *Lhx3-Peak-B* (ms) (chr2:26186472-26187246): Fwd-CAATGCAGGGTGACCTGG and Rev-GTGGGATTGACTGGGGTC. Hx-L-wt: ATTTGATTAATCA. ΔHx-L: AGCGGCCGCCTCA. *Isl1-Peak* (hum)(chr5:51559189-51559911): Fwd-CAGATGCACCTACCTCTTAAAG. Rev-GGACATATGGCTAGAGTGTGG. (1-409) Rev-CCCTACTCTGTCTGCCACTCC. Hx-S1-wt: TTTTAATTAGCT ΔHx-S1: TTTCTAGAAGCT. H2-wt: ATATTAAAAT ΔH2: ATCTAGAAAT. A/T motif-wt: AATTTTAGCATAT ΔA/T: ACGGTTGGCGCCT. *LMO4-Peak (ms)*(ch3:144198960-144199257): Fwd-GACGTGGGTCCCTTTAAGATCC and Rev-GGATTCTGCCTCCTCTCCTC. Hx-L-wt: AATTTTGTTAATTAA ΔHx-L: AACCATGGTAGGTAA.

### Immunofluorescence labeling

Embryos were fixed in 4% PFA/PBS for 90 min, embedded in OCT and cryosectioned at 12 µm. Embryos were incubated in primary antibody in either 0.1% fish gelatin or 0.3% bovine serum albumin (BSA) blocking buffer, overnight at 4°C. Primary antibodies used were goat (gt) anti-LacZ (Sigma 1:2000), rb anti-LacZ (Cappel 55976 1:2000), ms anti-Mnr2 (DSHB 5C10 1:250), and chicken (chk) anti-GFP (Aves Labs 1020 1:1000). Sections were imaged using a Zeiss Axio Imager.Z2 microscope.

### GFP quantification

Embryos used for GFP fluorescence analysis were not immunostained for GFP. A 750-ms exposure time was used for all images that were analyzed for GFP quantification. Integrative pixel density was measured in the ventral horn of the electroporated side of the spinal cord, using ImageJ. Four to twelve embryos were analyzed for each reporter construct. And for each embryo, average fluorescence intensity was calculated from analyzing three to seven sections.

### *In situ* hybridization

Embryos were electroporated with 1.75 µg/µL pBluescript expression vectors containing either mouse Isl1, rat Lhx3, or Isl1-Lhx3 fusion protein (Lee et al., 2012). Embryos were harvested at 3DPE and fixed in 4% PFA/PBS for 90 min. They were embedded in OCT and cryosectioned at 18 µm. Glassware for these experiments was treated with NaOH to avoid RNAase contamination. Sections were fixed in 4% PFA/PBS at room temperature for 10 min, then washed two times in PBS at room temp. Sections were then digested in proteinase K buffer (6.25mM EDTA, 0.05M Tris, and 1 µg/mL proteinase K) at room temperature for 5 min. Sections were fixed again in 4% PFA/PBS at room temperature for 5 min, and then washed two times in PBS at room temperature. Next, sections were submerged in 300-mL acetylation buffer (1.33% triethanolamine, 0.175% HCl). A total of 750 µL of acetic acid was gradually added to slides. Slides were incubated in acetic acid/acetylation buffer for 10 min at room temperature and then washed two times in PBS. Slides were then incubated in hybridization solution (0.75 M NaCl, 75 mM sodium citrate, 50% formamide, 5× Denhardt’s solution, and 1% herring sperm DNA) for 2 h at room temperature. To make *in situ* probes, cDNA for chick Lhx3, Isl1, and LMO4 3’ untranslated region (UTR) was cloned into pBluescript vector. Digoxigenin-labeled riboprobes were generated using T7 polymerase PCR. Probes were denatured in hybridization solution at 80°C for 5 min. Slides were incubated in probe/hybridization solution at 68°C overnight. Slides were then washed in 5× SSC (0.75 M NaCl and 75 mM sodium citrate) at 65°C for 10 min. Next, slides were incubated in 0.2× SSC (30 mM NaCl and 3 mM sodium citrate) at 65°C for 2 h and then washed in fresh 0.2× SSC at 65°C for 5 min. Slides were then blocked in buffer 1 (0.1 M Tris and 0.15 M NaCl) + 4% BSA at room temperature for 1 h, and then incubated in buffer 1 + 2% BSA + 1:5000 anti-digoxigenin antibody at 4°C overnight. Next, slides were washed three times with buffer 1 for 5 min each at room temperature, and then in buffer 2 (0.1 M Tris, 0.1 M NaCl, and 50 mM MgCl_2_) at room temperature for 5 min. Slides were then incubated in buffer 3 (0.1 M Tris, 0.1 M NaCl, 50 mM MgCl_2_, 2.4 µg/mL levamisole, 338 µg/mL NBT, and 175 µg/mL BCIP) at room temperature overnight, to perform the colormetric reaction. To stop the reaction, slides were then washed in TE. Prior to mounting, slides were dehydrated with serial ethanol washes (30%, 50%, 70%, 95%, and 100%) at room temperature and incubated in xylene for 10 min at room temp. Slides were then mounted using Permount.

### Luciferase assays

Assays were performed in cultured P19 embryonic mouse carcinoma cells. Cells were cultured in α-minimal essential media with 7.5% bovine calf serum and 2.5% fetal bovine serum. For luciferase assays, cells were seeded in 48 well plates, and transfected using Lipofectamine 2000 (Invitrogen). Cells were transfected with reporter constructs, transcription factor expression constructs, a CMV-β-galactosidase construct, to test transfection efficiency, and with empty plasmid to equalize the total amount of DNA for each condition. Luciferase and β-galactosidase activity was measured 48 h after transfection. Results are reported as activity fold change of each reporter construct when cotransfected with Isl1 plus Lhx3, compared to cotransfection with empty plasmid. Results from each experiment were obtained from the average of technical duplicates. Summarized results show the average activity fold change from at least five independent experiments.

## Results

### The Isl1-Lhx3 complex binds genomic loci associated with *Isl1*, *Lhx3*, and *Lmo4*

As transcriptional autoregulation is a powerful mechanism utilized by a variety of systems during development (Johnson et al., 1994; Belaguli et al., 1997; Smith et al., 2000; Aota et al., 2003; Bai et al., 2007; Borromeo et al., 2014), we hypothesized that the Isl1-Lhx3 complex may act to directly regulate its own expression. This hypothesis is further supported by the observation that the Isl1-Lhx3 fusion protein induces the transcription of *Isl1*, *Lhx3*, and *Lmo4* in the induced MN-embryonic stem cell (ESC) system (Lee et al., 2012). To test this hypothesis, we analyzed the previously reported data from ChIP experiments, performed in conjunction with high-throughput sequencing (ChIP-Seq) (Lee et al., 2013). These experiments used mouse MN-inducible (iMN)-ESCs, which have a doxycycline-inducible *Isl1-Lhx3* fusion gene. Dox treatment was coupled with a MN differentiation protocol to induce high levels of the Isl1-Lhx3 complex and MN differentiation (Lee et al., 2012, 2013).

**Figure 1.**
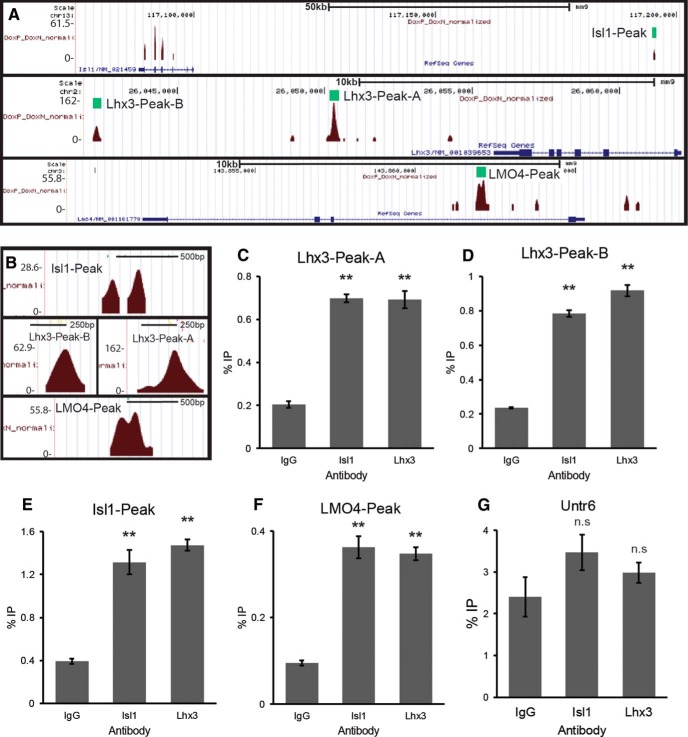
The Isl1-Lhx3 complex binds genomic loci associated with *Isl1*, *Lhx3*, and *Lmo4*. ***A***, Isl1-Lhx3 complex binding sites, identified via ChIP-Seq, in association with *Lhx3*, *Isl1*, and *Lmo4*. ***B***, A close-up of each ChIP-Seq peak. ***C–G***, E12.5 mouse spinal cord ChIP performed with Isl1 or Lhx3 antibodies. qPCR was performed for Lhx3-Peak-A, Lhx3-Peak-B, the Isl1-Peak, the LMO4-Peak, and the negative control region, Untr6. Experiments were performed independently three times. Results shown are from a single representative experiment; *n* = 3 technical replicates. Results were analyzed with a one-way ANOVA followed by Holm multiple comparison analysis. **p < 0.01, compared to nonspecific IgG controls. Error bars represent the SEM.

We found two Isl1-Lhx3 complex binding loci downstream of the *Lhx3* gene. Lhx3-Peak-A is located approximately 5.1 kb downstream of *Lhx3* (Fig. 1*A*). Lhx3-Peak-B is located approximately 19.5 kb downstream of *Lhx3* (Fig. 1*A*). We also found an Isl1-Lhx3 complex binding locus within a previously identified Isl1 enhancer (Isl1-Peak; Fig. 1*A*; Uemura et al., 2005; Kim et al., 2015). The Isl1-Lhx3 complex was also found to bind a locus within the first intron of *Lmo4* (LMO4-Peak; Fig. 1*A*). Given the known role of LMO4 in blocking the formation of the Lhx3 complex (Lee et al., 2008), this finding suggests an additional regulatory pathway to indirectly facilitate the formation of the Isl1-Lhx3 complex.

We assessed the in vivo occupancy of each peak by performing ChIP for endogenous Isl1 or Lhx3, using E12.5 mouse spinal cord lysates. We precipitated with either anti-Isl1, anti-Lhx3 or control IgG antibodies, followed by qPCR for Lhx3-Peak-A, Lhx3-Peak-B, the Isl1-Peak and the LMO4-Peak. Compared to IgG, both anti-Isl1 and anti-Lhx3 antibodies precipitated significantly more Lhx3-Peak-A, Lhx3-Peak-B, Isl1-Peak, and LMO4-Peak (Fig. 1*C–G*). As a negative control, we also performed qPCR for the untranslated genomic locus *Untr-6* (Mali et al., 2008) and saw no enrichment in the amount of *Untr-6* precipitated with anti-Isl1 or anti-Lhx3 antibodies, compared to IgG (Fig. 1*G*).

These results show that the Isl1-Lhx3 complex specifically binds Lhx3-Peak-A, Lhx3-Peak-B, the Isl1-Peak, and the LMO4-Peak during embryonic MN development in vivo. This finding supports a model in which these peaks serve as enhancers for the Isl1-Lhx3 complex to directly activate the transcription of *Lhx3*, *Isl1*, and *Lmo4*.

### Lhx3-Peak-A is activated by the Isl1-Lhx3 complex

**Figure 2.**
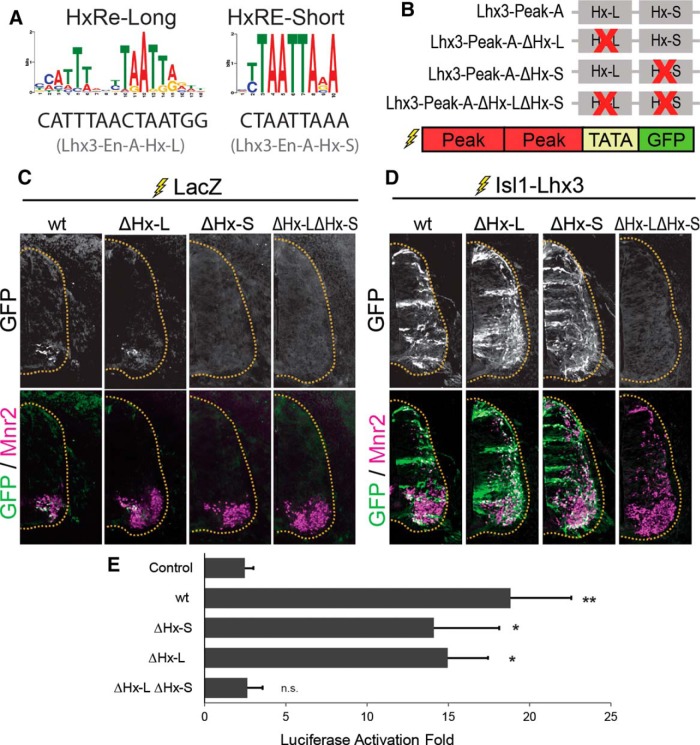
Lhx3-Peak-A is activated by the Isl1-Lhx3 complex. ***A***, HxRE-Long and HxRE-Short sequences identified by ChIP-Seq de novo motif analysis, and the HxRE-Long and HxRE-Short sequences in Lhx3-Peak-A. ***B***, Lhx3-Peak-A HxRE-Long and HxRE-Short mutants used for GFP-reporter experiments. ***C***, GFP-reporter experiments for Lhx3-Peak-A variants, embryos were electroporated with Lhx3-Peak-A-GFP reporter constructs plus ubiquitously expressing LacZ to mark electroporated cells. Sections were immunostained for GFP and Mnr2 to mark MNs. Images are representative of electroporations from multiple embryos. Lhx3-Peak-A-wt: *n* = 5, Lhx3-Peak-A-ΔHx-L: *n* = 14, Lhx3-Peak-A-ΔHx-S: *n* = 12, Lhx3-Peak-A-ΔHx-LΔHx-S: *n* = 15. ***D***, Embryos electroporated with Lhx3-Peak-A-GFP reporter construct plus Isl1-Lhx3 fusion protein construct. Sections were immunostained for GFP and Mnr2. Images are representative of electroporations from multiple embryos. Lhx3-Peak-A-wt: *n* = 25, Lhx3-Peak-A-ΔHx-L: *n* = 5, Lhx3-Peak-A-ΔHx-S: *n* = 13, Lhx3-Peak-A-ΔHx-LΔHx-S: *n* = 15. ***E***, Luciferase assays testing Lhx3-Peak-A wt and mutants. Lhx3-Peak variants are the same as those used in GFP-reporter experiments. Luciferase assays performed in cultured P19 cells. Results show the luciferase activation fold upon the addition of Isl1 plus Lhx3, compared to empty vector; *n* = 5 independent experiments. Results were analyzed with a one-way ANOVA followed by Holm multiple comparison analysis, comparing each reporter construct to control reporter (no enhancer). *p < 0.05, **p < 0.01. Error bars represent the SEM.

To test whether Lhx3-Peak-A activates transcription in MNs, we performed chick neural tube electroporation with a GFP-reporter construct containing two copies of Lhx3-Peak-A upstream of a minimally active TATA-box promoter and EGFP (Lhx3-Peak-A-GFP; Fig. 2*B*). Embryos were also electroporated with a ubiquitously expressing LacZ construct to mark electroporated cells. Chick embryos were electroporated at HH stage 14 and analyzed 3DPE. The expression of GFP from this reporter indicates the activation of Lhx3-Peak-A enhancer. We can further test the importance of a specific motif within the Lhx3-Peak-A enhancer by monitoring if mutation of the motif results in impaired expression of GFP.

Lhx3-Peak-A induced modest GFP expression, specifically in cells expressing the MN-specific gene Mnr2, a homolog of Hb9 (Fig. 2*C*). This result is consistent with the hypothesis that endogenous Isl1-Lhx3 complex activates *Lhx3* transcription in MNs via Lhx3-Peak-A. To test this directly, we coelectroporated Lhx3-Peak-A-GFP with an expression vector for Isl1-Lhx3 fusion protein. This construct activates ectopic expression of Isl1-Lhx3 fusion protein, which complexes with endogenous NLI to form the Isl1-Lhx3 complex (Lee et al., 2008; Song et al., 2009). Ectopic expression of the Isl1-Lhx3 complex activated GFP expression throughout the dorsal and ventral spinal cord (Fig. 2*D*). Further, both dorsal GFP^+^ cells and GFP^+^ cells in the ventral horn also expressed the MN-specific marker Mnr2 (Fig. 2*D*).

Because forced expression of the Isl1-Lhx3 complex initiates ectopic MN cell fate specification (Thaler et al., 2002; Lee et al., 2008, 2012), it was unclear whether the Isl1-Lhx3 complex directly activates GFP expression in ectopic MNs or whether the change in cell fate specification indirectly activates Lhx3-Peak-A. To test whether the Isl1-Lhx3 complex directly activates transcription via Lhx3-Peak-A, even without initiating MN fate specification, we performed luciferase reporter assays in cultured mouse embryonic carcinoma P19 cells. For these experiments, we transfected Lhx3-Peak-A-LUC reporters with expression vectors for Isl1, Lhx3, Isl1+Lhx3, or with empty vector. We cultured cells for 2 d and then performed luciferase assays to measure transcription of the luciferase reporter-gene. Transfection of Isl1 plus Lhx3 significantly activated Lhx3-Peak-A-LUC compared to control LUC reporter containing no enhancer (Fig. 2*E*). Combined with the ChIP-qPCR results from mouse embryonic spinal cord (Fig. 1*C*), these results show that the Isl1-Lhx3 complex directly binds Lhx3-Peak-A to initiate the transcription of *Lhx3*.

### Lhx3-Peak-A activity is mediated by two binding sites for the Isl1-Lhx3 complex

Lhx3-Peak-A contains both a putative HxRE-Long (Hx-L) motif and a putative HxRE-Short (Hx-S) motif (Fig. 2*A*). As both sequences are known binding sites of the Isl1-Lhx3 complex, we tested whether either or both contribute to Lhx3-Peak-A enhancer activity. To do this, we generated mutated versions of Lhx3-Peak-A where either the HxRE-Long, the HxRE-Short, or both sites are mutated (ΔHx-L, ΔHx-S, and ΔHx-LΔHx-S, respectively; Fig. 2*B*). Next, we made GFP-reporter constructs with each of these mutated versions of Lhx3-Peak-A and performed chick neural tube electroporation with either LacZ or Isl1-Lhx3.

When coelectroporated with LacZ, the ΔHx-L reporter construct still activated GFP expression in Mnr2-positive MNs (Fig. 2*C*). However, neither ΔHx-S, nor the double mutant activated any detectable GFP expression in the spinal cord (Fig. 2*C*). Coelectroporation with Isl1-Lhx3 activated both single mutant constructs throughout the spinal cord, but failed to activate the ΔHx-LΔHx-S double-mutant (Fig. 2*D*). These results show that both the HxRE-Long and the HxRE-Short in Lhx3-Peak-A contribute to its MN enhancer activity. Without the HxRE-Long, Lhx3-Peak-A is activated by endogenous levels of the Isl1-Lhx3 complex. However, when the HxRE-Short is ablated, the enhancer requires high levels of the Isl1-Lhx3 complex to be activated. And when both sites are mutated, the Isl1-Lhx3 complex cannot activate transcription via Lhx3-Peak-A.

We observed similar results with luciferase assays. In cells transfected with luciferase reporter constructs containing wt Lhx3-Peak-A, ΔHx-L, or ΔHx-S, transcription is activated by Isl1 plus Lhx3. However, when both response elements were mutated, cotransfection with Isl1 plus Lhx3 failed to activate transcription (Fig. 2*E*).

### Lhx3-Peak-B is activated in embryonic MNs

**Figure 3.**
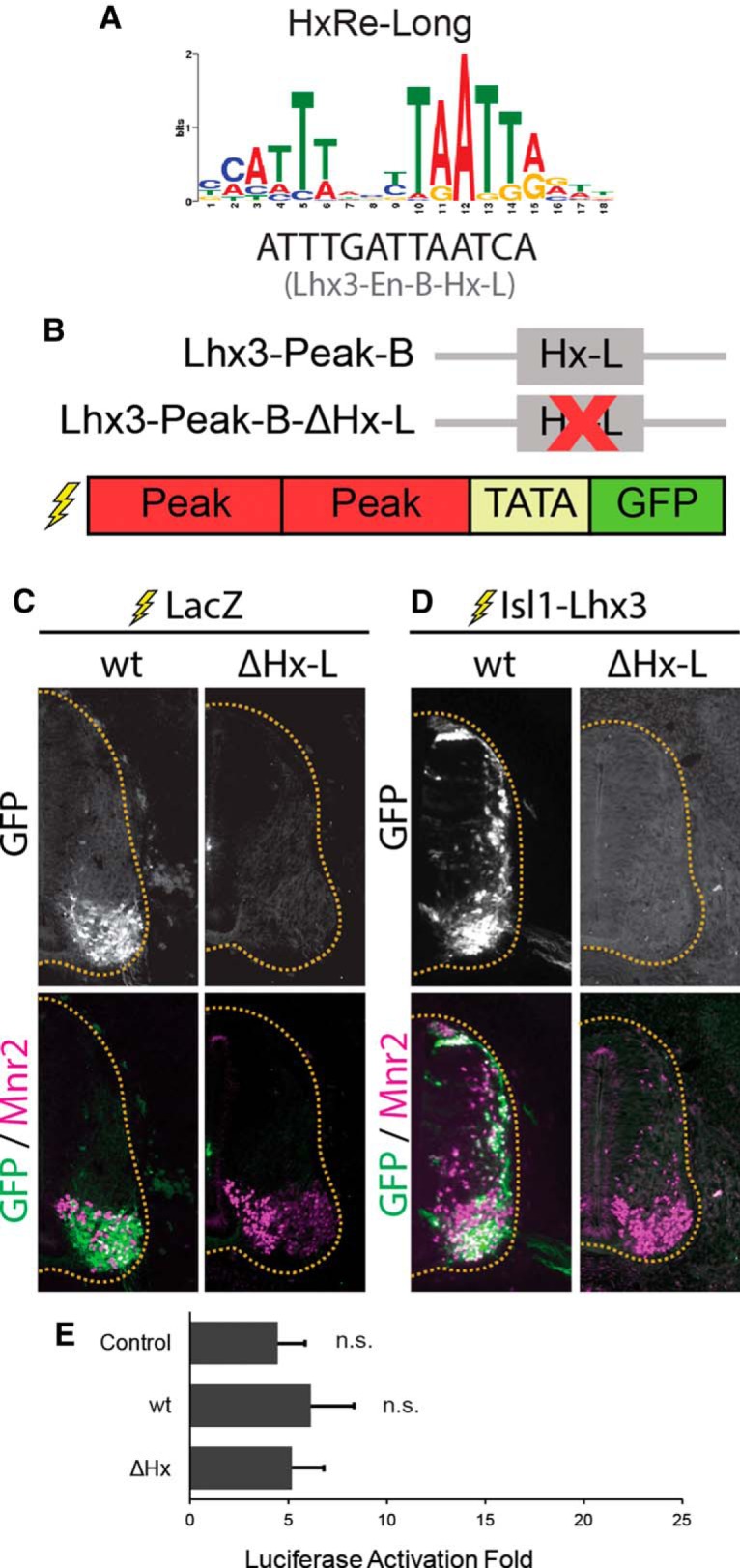
Lhx3-Peak-B is activated by the Isl1-Lhx3 complex. ***A***, HxRE-Long sequence identified by ChIP-Seq de novo motif analysis, and the HxRE-Long sequences in Lhx3-Peak-B. ***B***, Lhx3-Peak-B-wt and HxRE-Long mutant used for GFP-reporter experiments. ***C***, GFP-reporter experiments for Lhx3-Peak-B variants. Embryos were electroporated with Lhx3-Peak-B-GFP reporter constructs plus ubiquitously expressing LacZ to mark electroporated cells. Sections were immunostained for GFP and Mnr2 to mark MNs. Images are representative of electroporations from multiple embryos. Lhx3-Peak-B-wt: *n* = 20, Lhx3-Peak-B-ΔHx-L: *n* = 4. ***D***, Embryos electroporated with an Lhx3-Peak-B-GFP reporter construct plus Isl1-Lhx3 fusion protein construct. Sections were immunostained for GFP and Mnr2. Images are representative of electroporations from multiple embryos. Lhx3-Peak-B-wt: *n* = 7, Lhx3-Peak-B-ΔHx-L: *n* = 5. ***E***, Luciferase assays testing Lhx3-Peak-B wt and HxRE-Long mutant. Luciferase assays performed in cultured P19 cells. Results show the luciferase activation fold upon the addition of Isl1 plus Lhx3, compared to empty vector; *n* = 4 independent experiments. Results were analyzed with a one-way ANOVA followed by Holm multiple comparison analysis, comparing each reporter construct to control reporter (no enhancer). Error bars represent the SEM.

To test whether Lhx3-Peak-B acts as a MN-specific enhancer, we electroporated Lhx3-Peak-B-GFP with either LacZ or Isl1-Lhx3 in embryonic chick neural tube (Fig. 3*B*). When coelectroporated with LacZ, Lhx3-Peak-B activated GFP expression specifically and robustly in Mnr2-positive MNs (Fig. 3*C*). Coelectroporation of Lhx3-Peak-B-GFP with Isl1-Lhx3 activated GFP expression throughout the spinal cord, specifically in cells expressing ectopic or endogenous Mnr2 (Fig. 3*D*).

Interestingly, when we performed luciferase assays in P19 cells, Lhx3-Peak-B was not activated by cotransfection of Isl1 plus Lhx3, compared to control reporter construct with no enhancer (Fig. 3*E*). These results indicate that Lhx3-Peak-B acts as a strong MN-specific enhancer in embryonic MNs in vivo. However, the Isl1-Lhx3 complex is not sufficient to activate Lhx3-Peak-B in all cellular contexts. Cultured P19 cells could lack critical cofactors that are required for the Isl1-Lhx3 complex to activate transcription via Lhx3-Peak-B. However, as the Isl1-Lhx3 complex can activate other MN-specific enhancers in these cells, it is more likely that P19 cells express transcriptional repressors that specifically recognize Lhx3-Peak-B to block Isl1-Lhx3 complex binding or activity.

Lhx3-Peak-B contains one putative HxRE-Long motif (Fig. 3*A*). To test whether this motif contributes to the enhancer activity of Lhx3-Peak-B, we generated a mutated version of Lhx3-Peak-B where the HxRE-Long is mutated (ΔHx-L; Fig. 3*B*). When electroporated with LacZ, Lhx3-Peak-B-ΔHx-L-GFP did not activate any detectable GFP expression in Mnr2^+^ MNs. Coelectroporation of Isl1-Lhx3 fusion protein also failed to activate GFP expression (Fig. 3*D*), indicating that the HxRE-Long is critical for the MN-specific enhancer activity of Lhx3-Peak-B.

MN progenitor cells must rapidly upregulate the transcription of the Isl1-Lhx3 complex to promote terminal differentiation and MN cell fate specification. Immediately following the onset of Isl1 and Lhx3 expression in newly specified MNs, Lhx3-Peak-A and Lhx3-Peak-B likely contribute to this rapid increase in the transcription of *Lhx3.* This positive feedback-loop is expected to facilitate the switch from a nonspecified MN progenitor cell to a fully committed, differentiated MN.

### The Isl1-Peak is activated by the Isl1-Lhx3 complex via multiple HxRE motifs

**Figure 4.**
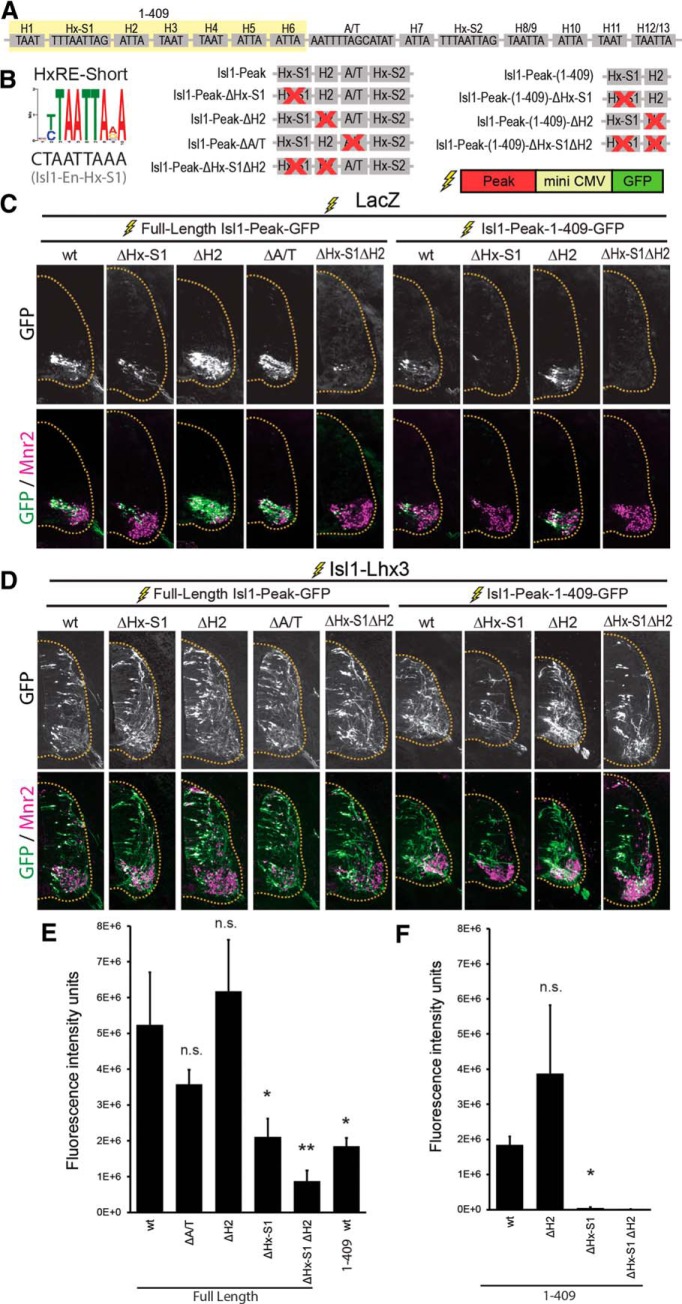
The Isl1-Peak is activated by the Isl1-Lhx3 complex. ***A***, Schematic representation of HxRE-S1, HxRE-S2, 13 TAAT motifs, and A/T-rich motif within the Isl1-Peak. Yellow shading indicates the sequences included in the shortened Isl1-Peak-(1-409). ***B***, HxRE-Short sequence identified by ChIP-Seq de novo motif analysis, and the HxRE-S1 sequences in Isl1-Peak. Isl1-Peak mutants used for GFP-reporter experiments. ***C***, GFP-reporter experiments for Isl1-Peak variants. Embryos were electroporated with Isl1-Peak-GFP reporter constructs plus ubiquitously expressing LacZ to mark electroporated cells. Sections were immunostained for Mnr2 to mark MNs. Images are representative of electroporations from multiple embryos. Isl1-Peak-wt: *n* = 5, Isl1-Peak-ΔHx-S1: *n* = 6, Isl1-Peak-ΔH2: *n* = 5, Isl1-Peak-ΔA/T: *n* = 16, Isl1-Peak-ΔHx-S1ΔH2: *n* = 6. Isl1-Peak-(1-409)-wt: *n* = 17, Isl1-Peak-(1-409)-ΔHx-S1: *n* = 10, Isl1-Peak-(1-409)-ΔH2: *n* = 9, Isl1-Peak-(1-409)-ΔHx-S1ΔH2: *n* = 20. ***D***, Embryos electroporated with an Isl1-Peak-GFP reporter construct plus Isl1-Lhx3 fusion protein construct. Sections immunostained for GFP and Mnr2. Images are representative of electroporations from multiple embryos. Isl1-Peak-wt: *n* = 6, Isl1-Peak-ΔHx-S1: *n* = 2, Isl1-Peak-ΔH2: *n* = 5, Isl1-Peak-ΔA/T: *n* = 3, Isl1-Peak-ΔHx-S1ΔH2: *n* = 4 . Isl1-Peak-(1-409)-wt: *n* = 5, Isl1-Peak-(1-409)-ΔHx-S1: *n* = 4, Isl1-Peak-(1-409)-ΔH2: *n* = 6, Isl1-Peak-(1-409)-ΔHx-S1ΔH2: *n* = 6. ***E***, ***F***, GFP fluorescence intensity for embryos electroporated with Isl1-Peak-GFP reporter constructs + LacZ; *n* = 4-12 embryos per condition. Results were analyzed with a one-way ANOVA followed by Holm multiple comparison analysis, comparing each mutant reporter construct to full-length wt-Isl1-Peak-GFP reporter or (***E***) wt-(1-409)-Isl1-Peak (***F***), *p < 0.05, **p < 0.01. Error bars represent the SEM.

The Isl1-Peak activates transcription in newly born MNs and in mature MMCm neurons in mouse, zebrafish and chick embryos (Uemura et al., 2005; Kim et al., 2015). We confirmed this finding in chick embryos by electroporating an Isl1-Peak-GFP reporter construct (Fig. 4*B*,*C*). It was also reported that the Isl1-Peak is activated by ectopic expression of the Isl1-Lhx3 complex (Kim et al., 2015). We tested this by electroporating Isl1-Peak-GFP with Isl1-Lhx3 fusion protein. We found that, indeed ectopic expression of the Isl1-Lhx3 complex expanded GFP expression to the dorsal spinal cord, and GFP expression colocalized with ectopic Mnr2 expression (Fig. 4*D*). These results are consistent with our findings that both Isl1 and Lhx3, bind to the Isl1-Peak in ESCs and in the mouse embryonic spinal cord (Fig. 1*A*,*E*).

The reported ChIP-Seq experiments (Lee et al., 2013) show that the Isl1-Peak contains two distinct Isl1-Lhx3 complex binding peaks (Fig. 1*B*), suggesting that there are at least two motifs regulating Isl1-Peak enhancer activity. The Isl1-Peak is 724 base pairs long. It contains motifs that are highly conserved between human and mouse, including 13 TAAT sites, and two sites that closely resemble HxRE-Short motifs (Hx-S1 and Hx-S2) (Fig. 4*A*,*B*). TAAT sequences act as binding sites for homeodomain transcription factors (H motifs; Gehring et al., 1994) and both the HxRE-Long and the HxRE-Short motifs contain TAAT sequences. Hx-S1 is located near the summit of the right peak of the Isl1-Peak (Fig. 1*B*), indicating that it may act as an Isl1-Lhx3 binding site. To test whether Hx-S1 contributes to the MN-specific enhancer activity of the Isl1-Peak, we electroporated Isl1-Peak-GFP reporter constructs with and without a mutation of the Hx-S1 motif (Fig. 4*B*). We found that, compared to wt-Isl1-Peak, Isl1-Peak-ΔHx-S1 activated significantly less GFP in embryonic MNs (Fig. 4*C*,*E*). To test whether Hx-S1 is sufficient for the Isl1-Lhx3 complex to activate Isl1-Peak, we also made GFP reporter constructs with a truncated version of the Isl1-Peak consisting of the first 409 nucleotides. This short version of the Isl1-Peak lacks the Hx-S2 motif [Isl1-Peak-(1-409); Fig. 4*B*]. In chick neural tube electroporations, Isl1-Peak-(1-409) activated GFP expression specifically in Mnr2 positive embryonic MNs, but significantly less effectively than full-length Isl1-Peak (Fig. 4*C*,*E*). GFP expression was further reduced when Hx-S1 was mutated in Isl1-En-(1-409)-GFP reporter experiments (Fig. 4*C*,*F*). These results suggest that, while Isl1-Peak-(1-409) is sufficient for the Isl1-Lhx3 complex to activate the Isl1-Peak via Hx-S1, other sequences within 410-724 nucleotides of the Isl1-Peak, such as the Hx-S2 motif, also contribute to its MN-specific enhancer activity.

Next, we coelectroporated each reporter construct with an Isl1-Lhx3 fusion protein vector to activate ectopic expression of the Isl1-Lhx3 complex. Surprisingly, we found that, like the wt Isl1-Peak, each mutant Peak, including Isl1-Peak-(1-409)-ΔHx-S1, activated robust GFP in the dorsal spinal cord that colocalized with ectopic Mnr2 expression (Fig. 4*D*). Notably, this approach can detect even weaker enhancer activity due to expression of high levels of the Isl1-Lhx3 complex. Therefore, Isl1-Peak-(1-409) likely contains additional motifs that, in the absence of Hx-S1, are not active in Mnr2-positive embryonic MNs but respond to high levels of the Isl1-Lhx3 complex. In support of this hypothesis, many H motifs in Isl1-Peak show some homology to either HxRE-Long or HxRE-Short motif. For instance, H2 shows weak homology to the HxRE-Short motif. To test whether H2 can independently respond to the Isl1-Lhx3 complex, we constructed Isl1-Peak-GFP and Isl1-Peak-(1-409)-GFP constructs with a mutation in H2 alone or combined with the ΔHx-S1 mutation. While mutation of H2 alone did not reduce GFP expression (Fig. 4*C*,*E*,*F*), mutating both Hx-S1 and H2 in the full-length Isl1-En caused a slight reduction in GFP expression compared to mutating Hx-S1 alone (Fig. 4*C*,*E*). This trend was not statistically significant, but raises the interesting possibility that the H2 motif could function as a cryptic HxRE motif, which manifests its activity only in the absence of Hx-S1 motif. Hx-S1 clearly contributes substantially to the enhancer activity of the Isl1-Peak, and it appears that H2 may also facilitate Isl1-Peak activation in embryonic MNs. Coelectroporation of Isl1-Peak-(1-409)-ΔHx-S1ΔH2-GFP and Isl1-Lhx3 activated robust GFP in the dorsal spinal cord (Fig. 4*D*), suggesting that additional H motifs similarly function as cryptic HxRE motif(s) to activate the Isl1-Peak.

We also mutated the A/T-rich motif located at 470 base pairs (ΔA/T), which is only present in the full-length version of the Isl1-Peak (Fig. 4*A*,*B*). Previous reports have shown that this site is required for Isl1-Peak enhancer activity in endogenous MNs and in ectopic MNs induced by overexpression of the Isl1-Lhx3 complex (Kim et al., 2015). However, we found that the ΔA/T mutant activates robust GFP expression in both endogenous and ectopic MNs (Fig. 4*C*,*D*). In endogenous MNs, there was no difference between ΔA/T-GFP expression and wt Isl1-Peak-GFP expression, indicating that this motif does not contribute to Isl1-Peak enhancer activity (Fig. 4*E*).

Overall, these findings support a model in which the Isl1-Lhx3 complex activates the Isl1-Peak via HxRE-S1, likely in cooperation with additional HxRE and H motifs. These results are also consistent with our findings that both Isl1 and Lhx3, bind to the Isl1-Peak in ESCs and in the mouse embryonic spinal cord (Fig. 1*A*,*E*).

### The LMO4-Peak is activated by the Isl1-Lhx3 complex via a single HxRE-Long motif

**Figure 5.**
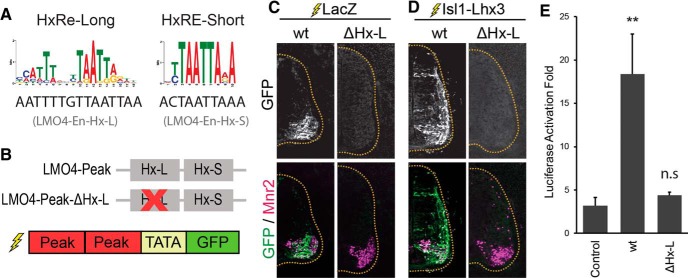
The LMO4-Peak is activated by the Isl1-Lhx3 complex. ***A***, HxRE-Long and HxRE-Short sequences identified by ChIP-Seq de novo motif analysis, and the HxRE-Long and HxRE-Short sequences in the LMO4-Peak. ***B***, LMO4-Peak HxRE-Long mutant used for GFP-reporter experiments. ***C***, GFP-reporter experiments for LMO4-Peak variants. Embryos were electroporated with LMO4-Peak-GFP reporter constructs plus ubiquitously expressing LacZ to mark electroporated cells. Sections were immunostained for GFP and Mnr2 to mark MNs. Images are representative of electroporations from multiple embryos. LMO4-Peak-wt: *n* = 13, LMO4-Peak-ΔHx-L: *n* = 12. ***D***, Embryos electroporated with LMO4-Peak-GFP reporter construct plus Isl1-Lhx3 fusion protein construct. Sections were immunostained for GFP and Mnr2. Images are representative of electroporations from multiple embryos. LMO4-Peak-wt: *n* = 4, LMO4-Peak-ΔHx-L: *n* = 5. ***E***, Luciferase assays testing LMO4-Peak-wt and mutants. Luciferase assays performed in cultured P19 cells. Results show the luciferase activation fold upon the addition of Isl1 plus Lhx3, compared to empty vector; *n* = 5 independent experiments. Results were analyzed with a one-way ANOVA followed by Holm multiple comparison analysis, comparing each reporter construct to control reporter (no enhancer). **p < 0.01. Error bars represent the SEM.

During MN specification, LMO4 blocks the formation of the V2-interneuron specifying Lhx3 complex, and thereby inhibits the expression of V2-specific genes in MNs (Lee et al., 2008). In addition to rapidly and robustly upregulating its own expression, we hypothesized that the Isl1-Lhx3 complex also activates the transcription of LMO4 in newly differentiating embryonic MNs. To test whether the LMO4-Peak (Fig. 1*A*) acts as an enhancer in embryonic MNs, we performed chick neural tube electroporations with an LMO4-Peak-GFP reporter construct (Fig. 5*B*). When we electroporated LMO4-Peak-GFP with LacZ, we found that the LMO4-Peak activates GFP expression specifically in Mnr2^+^ MNs (Fig. 5*C*). Coelectroporating Isl1-Lxh3 with LMO4-Peak-GFP, expanded GFP expression throughout the spinal cord, specifically in cells expressing endogenous or ectopic Mnr2 (Fig. 5*D*).

Luciferase assays using LMO4-Peak-LUC with Isl1, Lhx3, or Isl1 plus Lhx3, were consistent with these results (Fig. 5*E*). Isl1 plus Lhx3 significantly activated LMO4-Peak-LUC expression, compared to control vector containing no enhancer (Fig. 5*E*). These results indicate that, in embryonic MNs, the LMO4-Peak recruits the Isl1-Lhx3 complex to activate the transcription of *Lmo4*. By blocking the formation and activity of the Lhx3 complex, LMO4 inhibits the transcription of V2-IN specific genes in MNs and increases the pool of free Lhx3 available to incorporate into the Isl1-Lhx3 complex.

The LMO4-Peak contains one HxRE-Long motif and one HxRE-Short motif (Fig. 5*A*). To test whether the HxRE-Long motif contributes to the activity of the LMO4-Peak, we generated a mutated version of the LMO4-Peak where the HxRE-Long sequence is ablated (ΔHx-L; Fig. 5*B*). Chick neural tube electroporations with ΔHx-L-GFP did not activate any detectable GFP expression in the embryonic spinal cord (Fig. 5*C*). Coelectroporation of Isl1-Lhx3 fusion protein with the mutated reporter also failed to activate GFP expression (Fig. 5*D*). Likewise, ΔHx-L-LUC was not activated by cotransfection with Isl1 plus Lhx3 in P19 cells (Fig. 5*E*). These results indicate that the HxRE-Long motif is required for the Isl1-Lhx3 complex to activate transcription via the LMO4-Peak.

### The Isl1-Lhx3 complex activates the transcription of endogenous Lhx3, Isl1, and LMO4

To test whether the Isl1-Lhx3 complex can activate the transcription of *Isl1*, *Lhx3*, and *Lmo4*, in the embryonic spinal cord, we ectopically expressed mouse Isl1 and rat Lhx3, or Isl1-Lhx3 fusion protein in the embryonic chick spinal cord through neural tube electroporation. We harvested embryos at 3DPE and performed *in situ* hybridizations with chk-specific probes designed to recognize the 3’UTR of chick *Isl1*, *Lhx3*, or *Lmo4*. Because the Isl1, Lhx3, and Isl1-Lhx3 expression constructs lack 3’UTR sequences, these probes exclusively detect endogenous chick transcripts.

**Figure 6.**
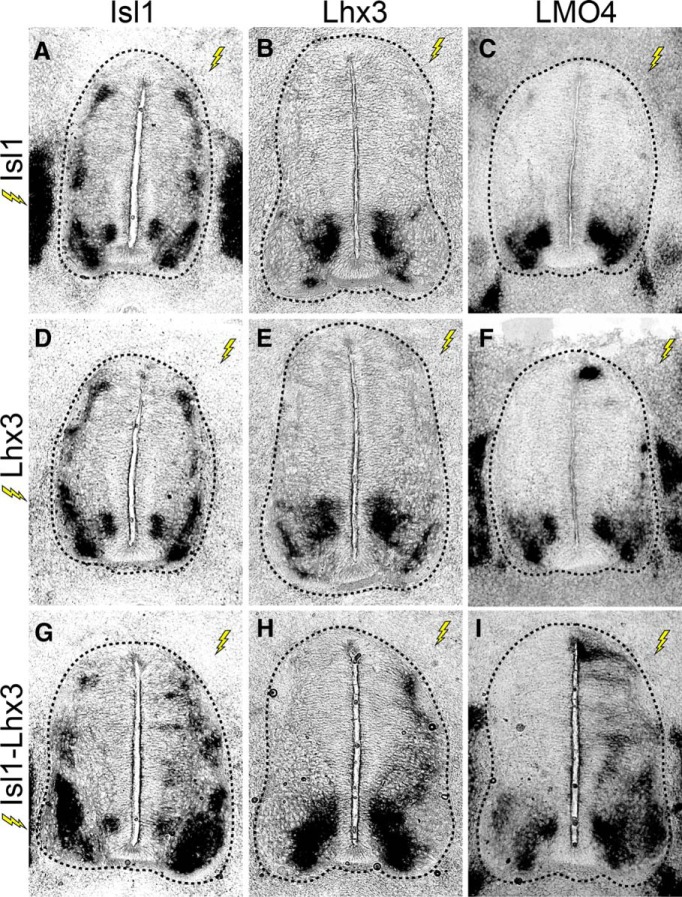
The Isl1-Lhx3 complex activates transcription of endogenous Isl1, Lhx3, and Lmo4. ***A–I***, Embryos were electroporated with Isl1, Lhx3, or Isl1-Lhx3. *In situ* hybridization shows the transcription of endogenous *Isl1*, *Lhx3*, or *Lmo4*. Lightning bolts indicate the electroporated side of the embryo (right side), compared to the unelectroproated, control side (left side).

Embryos that were electroporated with Isl1 alone showed no change in the expression of endogenous *Isl1*, *Lhx3*, or *Lmo4* (Fig. 6*A--C*). Lhx3 electroporation slightly increased the transcription of *Lmo4*, but did not affect expression of endogenous *Isl1* or *Lhx3* (Fig. 6*D–F*). In contrast, embryos that were electroporated with Isl1-Lhx3 showed robust increases in transcription of *Isl1*, *Lhx3*, and *Lmo4* throughout the dorsal spinal cord (Fig. 6*G–I*). These results show that the Isl1-Lhx3 complex is sufficient to induce the transcription of *Lhx3*, *Isl1*, and *Lmo4* in the embryonic spinal cord.

## Discussion

A great deal of progress has been made characterizing the activity and expression patterns of Isl1, Lhx3, and LMO4 in embryonic MNs (Thaler et al., 2002; Lee et al., 2008, 2012, 2013; Rousso et al., 2008; Song et al., 2009; Roy et al., 2012). However, the mechanisms that activate the transcription of these factors in differentiating MNs, and the pathways that regulate their expression in specific MN subtypes remain unclear. Our results show that the Isl1-Lhx3 complex binds genomic loci associated with *Lhx3*, *Isl1*, and *Lmo4*, both in an iMN-ESC system, and in the embryonic spinal cord. Each of these loci acts as a MN-specific enhancer and is robustly activated by the Isl1-Lhx3 complex. Additionally, we show that overexpression of the Isl1-Lhx3 complex activates the transcription of endogenous *Isl1*, *Lhx3*, and *Lmo4*. Together, these results show that, early in embryonic MN specification, the Isl1-Lhx3 complex is recruited to loci associated with *Isl1*, *Lhx3*, and *Lmo4* to directly activate the transcription of each of these genes. This transcriptional activation generates a positive autoregulatory feedback loop where the Isl1-Lhx3 complex activates the transcription of its own components. This feedback loop contributes to the rapid induction of Isl1, Lhx3, and LMO4 expression in differentiating MNs, and to the maintenance of these factors in mature MMCm neurons.

### Expression of the Isl1-Lhx3 complex

Onecut transcription factors, including Hnf6, activate the transcription of Isl1 in early MNs and regulate the expression of Isl1 in multiple MN subtypes (Roy et al., 2012). However, in the absence of Hnf6 and Onecut-2, newly generated MNs still maintain low levels of Isl1 expression, and normal numbers of MNs are generated, indicating that there are additional pathways contributing to the onset of Isl1 expression (Roy et al., 2012). Likewise, the activation of Lhx3 and LMO4 expression in differentiating MNs is critical for MN specification, and little is known regarding the specific mechanisms that regulate the expression of these two factors (Sharma et al., 1998; Lee et al., 2008).

Early in MN specification, Hb9 is released from transcriptional repression via reduced levels of Olig2 expression (Lee et al., 2005). Reduced Olig2 expression could also release Isl1 and Lhx3 from transcriptional repression, which would allow for modest expression of Isl1 and Lhx3. Our results in this report suggest that the resulting low levels of the Isl1-Lhx3 complex, at the onset of MN specification, activates a positive transcriptional feedback loop that rapidly induces high levels of Isl1 and Lhx3 expression. At the same time, the Isl1-Lhx3 complex concurrently activates *Lmo4* transcription. LMO4 competes with Lhx3 to bind NLI, thereby blocking the formation of the Lhx3 complex. This action inhibits the transcription of V2-interneuron genes in MNs and increases the available pool of Lhx3, which indirectly promotes Lhx3 incorporation into the Isl1-Lhx3 complex.

Positive and negative transcriptional feedback loops, both direct and indirect, have been shown to contribute to the temporal regulation of gene expression in a variety of cellular contexts (Harris and Levine, 2005; Haberland et al., 2007; Svenningsen et al., 2008; DiTacchio et al., 2012; Morichika et al., 2012; Pruunsild et al., 2013; Bornstein et al., 2014). In particular, transcriptional autoregulation is prominent in development and cell specification (Johnson et al., 1994; Belaguli et al., 1997; Smith et al., 2000; Aota et al., 2003; Bai et al., 2007; Borromeo et al., 2014). Positive autoregulation of the Isl1-Lhx3 complex is an efficient mechanism to ensure the rapid transition from a pluripotent, progenitor cell state to a post-mitotic, differentiated MN. It facilitates rapid induction of the Isl1-Lhx3 complex and thereby, quickly induces the expression of genes essential for MN differentiation such as *Hb9* (Arber et al., 1999).

### Recruitment of the Isl1-Lhx3 complex

While each peak in this study is activated by the Isl1-Lhx3 complex, the composition of each peak varies. Thus, the genetic mechanisms utilized to recruit the Isl1-Lhx3 transcription complex also vary. Both Lhx3 peaks and the LMO4 peak contain single HxRE-Long motifs, while Lhx3-Peak-A and the LMO4-Peak also contain single HxRE-Short motifs. The Isl1-Peak contains two HxRE-Short motifs, but no obvious HxRE-Long motifs, as well as multiple H motifs that resemble HxRE-Long and HxRE-Short motifs.

The enhancer activity of Lhx3-Peak-A is only completely lost when both the HxRE-Long and HxRE-Short motifs are ablated. This finding shows that these two motifs cooperate to recruit the Isl1-Lhx3 complex and induce transcriptional activation. In contrast to this observation, the HxRE-Long motifs in Lhx3-Peak-B and the LMO4-Peak are critical for the activity of these enhancers. When the HxRE-Long motifs in Lhx3-Peak-B or the LMO4-Peak are ablated, neither is responsive to even high levels of ectopic Isl1-Lhx3 complex expression. This result is consistent with Lhx3-Peak-B containing no additional HxRE motifs. However, unlike Lhx3-Peak-A, the HxRE-Short motif in the LMO4-Peak is unable to compensate for the loss of the HxRE-Long motif. Further mutational analysis of the LMO4-Peak, in which the HxRE-Short is ablated, will be necessary to determine whether this site can also contribute to the activity of this enhancer.

Unlike the Lhx3 and LMO4 peaks, we found that the activity of the Isl1-Peak is mediated by cooperative action of multiple motifs. Hx-S1 contributes substantially to Isl1-Peak enhancer activity, but ablating Hx-S1 is not sufficient to completely abolish its enhancer activity. These findings indicate that other motifs, possibly HxRE-S2 and H motifs also contribute to the activity of the Isl1-Peak. As demonstrated by H2 (Fig. 4*E*), some motifs may function as alternative binding sites for the Isl1-Lhx3 complex only in the absence of Hx-S1 or cooperate with Hx-S1 to recruit the Isl1-Lhx3 complex in developing MNs. Further study of this unique enhancer could reveal interesting genetic mechanisms to refine transcriptional specificity.

Lee et al. (2008) has previously reported that HxRE-Short motifs also serve as high affinity binding sites for the V2-IN-specifying Lhx3 complex. We have also shown that, in embryonic MNs, Hb9 recognizes and binds HxRE-Short motifs to inhibit the transcription of an Lhx3 complex target gene *Chx10* (Lee et al., 2008). Our results indicate that the HxRE-Short motifs found in Lhx3-Peak-A, the Isl1-Peak and the LMO4-Peak are likely not recognized by Hb9. This finding suggests that Hb9 binds only a subset of high affinity HxRE-Short motifs. This is an interesting hypothesis that raises questions regarding the specificity of Hb9 binding during embryonic MN development. Future genome-wide analysis of Hb9 binding sites in embryonic MNs will provide critical insight into this issue.

### Isl1 and Lhx3 expression in MN subtypes

In addition to facilitating Isl1 and Lhx3 transcription during MN specification, the Isl1-Peak and Lxh3-Peaks that we have characterized in this study likely act to maintain high levels of Isl1 and Lhx3 expression in mature MMCm neurons. Following MN specification, many MN subtypes downregulate the expression of *Isl1* or *Lhx3*. LMCl neurons do not express *Isl1*, and *Lhx3* expression is only maintained in MMCm neurons (Tsuchida et al., 1994; Rousso et al., 2008). To halt the expression of Isl1 or Lhx3, MNs must disrupt the positive transcriptional feedback loop generated by these proteins. Transcriptional repressor proteins or translational repressing pathways, such as the expression of specific micro-RNAs, would be efficient mechanisms to downregulate the expression of Isl1 or Lhx3. While a great deal of work has been done to characterize the genetic mechanisms that activate the expression of specific transcription factors and signaling molecules during MN-subtype development, the pathways utilized to repress specific genes are not well understood. These repressive pathways are critical for MN-subtype development, as forced expression of Lhx3 has been shown to convert MNs to an MMCm fate (Sharma et al., 2000). It will therefore be important to identify the mechanisms utilized to downregulate Isl1 and Lhx3 expression in specific MN subtypes and to determine whether such mechanisms interrupt the positive autoregulatory pathways defined in this study, to build a comprehensive model of transcriptional regulation in MN development.

## Synthesis

Reviewing Editor: Esther Stoeckli, University of Zurich Decisions are customarily a result of the Reviewing Editor and the peer reviewers coming together and discussing their recommendations until a consensus is reached. When revisions are invited, a fact-based synthesis statement explaining their decision and outlining what is needed to prepare a revision will be listed below. The following reviewer(s) agreed to reveal their identity: Artur Kania, Benjamin Deneen.

We have discussed your revised manuscript and have agreed that the manuscript is essentially acceptable without further experiments. However, a few things need to be taken care of before we can accept the paper for publication:

We noticed that you used the Student's t-test throughout. This is not appropriate when you compare more than two conditions/groups. Instead ANOVA should be used for the statistical analysis. Please recalculate the statistics. Furthermore, we would also like to see a description of the in situ hybridization protocol added to the Material & Method section. And finally, please use a more readable font for the figures. It may help to not use BOLD font for the labels. This is particularly important for Figure 2B, 3B, 4A, 4B, 4C, 4D, 5B. Usually Arial or Helvetica is easy to read.
